# Perioperative management of colorectal surgical patients receiving a direct oral anticoagulant: a scoping review, particular emphasis on procedure-specific risks, and pharmacogenomics

**DOI:** 10.3389/fcvm.2026.1626969

**Published:** 2026-02-26

**Authors:** Jieling Mao, Li Qin, Min Gao, Jingwen Xie, Xiaoyan Li, Zhikun Liang

**Affiliations:** 1 Department of Pharmacy, The Sixth Affiliated Hospital, Sun Yat-Sen University, Guangzhou, China; 2 School of Pharmaceutical Science, Sun Yat-Sen University, Guangzhou, China; 3 Biomedical Innovation Center, The Sixth Affiliated Hospital, Sun Yat-Sen University, Guangzhou, China

**Keywords:** bleeding, colorectal surgery, direct oral anticoagulants, pharmacodynamic, pharmacokinetic, venous thromboembolism

## Abstract

Perioperative management of patients on direct oral anticoagulants (DOACs) for preoperative deep vein thrombosis (DVT), pulmonary embolism (PE), or atrial fibrillation (AF), who subsequently undergo elective colorectal surgery, is a frequent clinical scenario with no clear consensus on best practices. Further complicating this issue, venous thromboembolism (VTE) and bleeding rates vary widely, ranging from 4.8% to 12.6% for VTE and 1.1% to 2.4% for bleeding, across different procedures (e.g., abdominoperineal resection, anterior resection, rectopexy, colectomy, and total proctocolectomy), as well as between countries, centers and individual surgeons. Therefore, it is necessary for surgeons to identify strategies to optimize when and how to discontinue and resume anticoagulation. Over the past decade, substantial interpatient variability in DOAC plasma levels has been observed, potentially explaining the frequent incidence of clinically relevant nonmajor bleeding (e.g., anastomotic bleeding and hematochezia) and breakthrough VTE in colorectal surgical patients. Given that pharmacokinetic factors, including genetic variations in metabolizing enzymes and efflux transporters as well as drug plasma levels measured by anti-factor Xa (FXa) activity, are associated with both the efficacy and adverse effects of anticoagulants, genotyping and anti-FXa monitoring could play a valuable role in optimizing perioperative DOAC management or enabling personalized dose adjustments. This scoping review summarizes the current evidence and proposes an integrated, personalized approach for perioperative DOAC management in colorectal surgery, with particular emphasis on procedure-specific risks, pharmacogenomics, and individualized risk prediction.

## Introduction

Colorectal surgery is one of the most common operations worldwide. It is estimated that annually, 15%–20% of patients on chronic anticoagulation require elective colorectal surgery or procedures, presenting a common clinical dilemma in managing perioperative anticoagulation (1). Anticoagulation can be a double-edged sword as it increases the risk of bleeding, particularly in colorectal surgical patients. Decisions regarding the interruption, bridging, and resumption of anticoagulants during the perioperative period need to be individualized. However, venous thromboembolism (VTE) occurs in up to 12.6% of patients undergoing colorectal surgery (2), and bleeding rates vary widely across different procedures, further complicating use of anticoagulation in this population (3).

Although direct oral anticoagulants (DOACs) have predictable pharmacokinetics (PK) and pharmacodynamics (PD), allowing fixed dosing without routine coagulation monitoring, recent reports highlight significant interindividual variability in plasma levels and drug responses, which may increase the risk of bleeding or thromboembolism (4). Given that PK factors, including genetic variations in metabolizing enzymes and efflux transporters as well as drug plasma levels indicated by anti-factor Xa (FXa) activity, are associated with the efficacy and adverse effects of anticoagulants (5), genotyping and anti-FXa monitoring could play a valuable role in optimizing perioperative DOAC management or enabling personalized dose adjustments.

**Figure 1:**
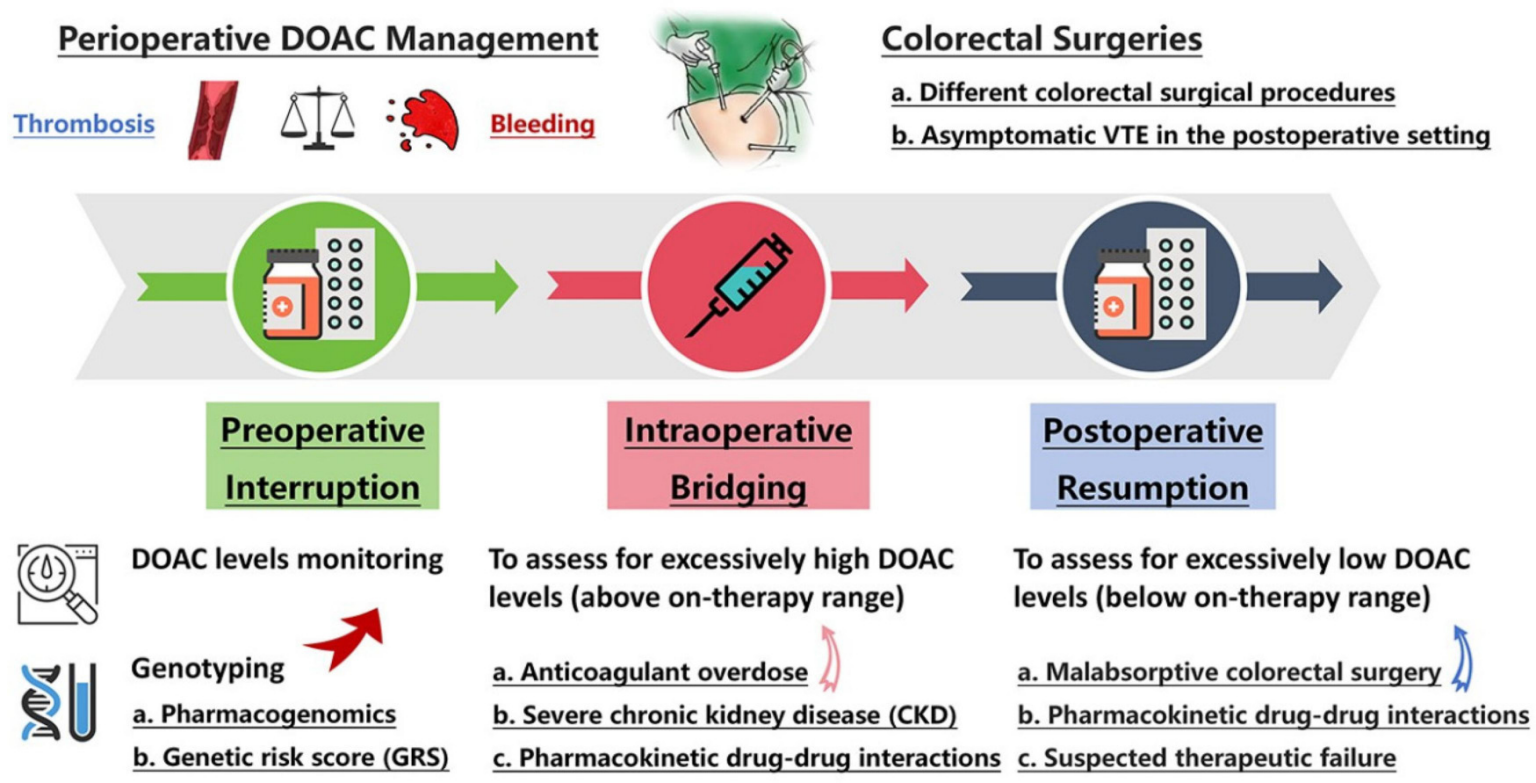
Perioperative management of DOAC for patients undergoing colorectal surgery. CKD, chronic kidney disease; DOAC, direct oral anticoagulants; GRS, genetic risk score; VTE, venous thromboembolism.

Here, this review synthesizes evidence to provide optimal perioperative management strategies for DOAC in patients undergoing elective colorectal surgery (Figure 1). Specifically, we integrate recent large-scale, procedure-specific data on VTE and bleeding risks, assess the emerging role of pharmacogenomics in personalizing therapy, and incorporate real-world evidence from diverse populations. This review systematically examines the interindividual variability in DOAC plasma levels, the pharmacogenomic information influencing interindividual variability, and the roles of genotyping and anti-FXa monitoring in perioperative DOAC management and individualized dose adjustment. It should be noted that certain clinical scenarios such as heparin-induced thrombocytopenia (HIT), recent coronary revascularization, and patients with documented antiphospholipid syndrome or inherited thrombophilia are beyond the scope of this review.

## Methods

This review is based on a comprehensive literature search conducted in PubMed, Embase, and Web of Science databases from January 2017 to March 2025, using keywords including “direct oral anticoagulants”, “colorectal surgery”, “perioperative management”, “pharmacogenomics”, “venous thromboembolism” and “bleeding”. Studies were screened based on relevance on elective colorectal surgery and DOAC use, with priority given to RCTs, meta-analyses, and large cohort studies. Additional references were retrieved from the bibliographies of key articles.

### Study quality assessment

We assessed the quality of the included evidence using the GRADE (Grading of Recommendations, Assessment, Development, and Evaluations) framework. GRADE is a widely adopted, transparent system for evaluating evidence and formulating clinical recommendations. It classifies the certainty of evidence as high, moderate, low, or very low based on explicit criteria across five key domains: risk of bias, inconsistency, indirectness, imprecision, and publication bias. Applying these criteria, the overall evidence synthesized in this review was judged to be of moderate or high certainty.

## Different colorectal surgical procedures and their associated rates of VTE and bleeding

Colorectal surgery plays a vital role in the management of various diseases, including colorectal cancer (CRC), inflammatory bowel disease (IBD), diverticular disease, and other colorectal disorders. The choice of procedures depends on the specific condition, tumor location, and patient factors (6). The risk of postoperative VTE and bleeding is influenced by factors such as prolonged surgery duration, patient immobility, and underlying medical conditions (7, 8). Postoperative VTE and bleeding rates are widely reported quality metrics and used in pay-for-performance programs in medical institutions of different levels. Given that postoperative VTE is often asymptomatic, the reported incidence is subject to surveillance bias (9). In the aspect of postoperative bleeding, gastrointestinal bleeding (e.g., rectal bleeding, melena, and hematemesis) and anastomotic bleeding are usually symptomatic in clinical practice. As a result, the rates of VTE vary considerably, depending on the screening protocol, while bleeding rate are relatively stable.

### Abdominoperineal resection

Abdominoperineal resection (APR) for rectal cancer involves removing the rectum and anus and creating an end colostomy (10). The procedure is technically demanding due to the pelvic anatomy, with notable risks of presacral venous plexus hemorrhage and injury to adjacent structures such as the vagina, bladder, prostate, ureters, and pelvic nerves (11). Additionally, APR is associated with a high complication rate, as consistently reported in the literature. Specifically, for open APR, the risk of symptomatic VTE at 4 wk was 3.6%, and the risk of bleeding requiring transfusion was 21.5% (12). In contrast, for laparoscopic APR, the risk of symptomatic VTE was 1.1%, and the risk of bleeding requiring transfusion was 4.9% (12).

### Anterior resection

For rectal cancer sparing the anal sphincter, anterior resection, or abdominal proctosigmoidectomy, is the indicated surgical procedure (13). It entails the excision of the rectosigmoid segment and the subsequent anastomosis of the descending colon to the proximal rectum. This sphincter-preserving method, which precludes perineal dissection, thereby maintains intestinal continuity and optimizes postoperative functional outcomes. Sphincter-preserving surgery in the form of low anterior resection (LAR) is recognized as the gold standard for localized rectal cancer (14). LAR accounts for up to 80% of rectal cancer procedures (15). In 2023, a meta-analysis incorporating 18 clinical studies on anterior resection showed that for minimally invasive anterior resection, the risk of symptomatic VTE ranged from 0.8% to 3.2%, with a clinical relevant bleeding risk of 2.4%. For open anterior resection, the risk of symptomatic VTE was between 1.0% and 4.0% (3).

### Colectomy and total proctocolectomy

Colectomy involves the removal of part or all of the colon and can be performed for CRC. The decision to perform segmental or extended colectomy in CRC patients must consider the risk of metastasis and functional consequences of the surgery, age, and patients' wishes (16). The procedure can be done through open surgery or minimally invasive techniques such as laparoscopic or robotic-assisted surgery. The risk of symptomatic VTE is between 1.8% and 3.4%. The risk of VTE also varies by extent of bowel resection: minimally invasive and open left (1.4%) and right (1.9%) hemicolectomies have lower VTE risk compared with total proctocolectomies or total colectomies (laparoscopic 5.0% and open 5.4%) (3). As for bleeding requiring reintervention, minimally invasive right colectomy has the highest risk (1.5%), followed by minimally invasive colectomy for malignant disease (1.3%) (3).

Although patients with IBD are usually treated medically, surgery is required in patients who develop severe complications and those who are refractory to medical therapy (17). Subtotal colectomy with or without ileal pouch-anal anastomosis (IPAA) can be performed on IBD patients (18). The risk of symptomatic VTE is slightly higher than that of malignant disease with rates from 2.1% to 4.1% (3). Postoperative bleeding from the remnant rectum is a procedure-specific complication after subtotal colectomy (19). The reported incidence of postoperative bleeding from the remnant rectum ranges from 1.8% to 8.2% (19). Total proctocolectomy (TPC) is the surgical resection of the entire colon and rectum with or without perineal dissections (20). This procedure is associated with a higher risk of symptomatic VTE due to its extensive nature and the potential for postoperative complications (21). The reported VTE incidence ranges from 4.3% to 12.6%, while clinical relevant bleeding rates can reach up to 2.4%. The complexity of the surgery and the need for careful anastomosis contribute to these risks.

### Rectopexy

Rectopexy is a surgical procedure used to treat rectal prolapse by securing the rectum to the sacral promontory (22). This procedure can be performed through an abdominal or perineal approach. Abdominal surgery for rectal prolapse requires one larger incision or multiple smaller incisions (23). The risk of VTE in rectopexy is relatively lower compared to more extensive colorectal procedures (24). It has been reported that within 30 days post-operation, the incidence of symptomatic VTE for laparoscopic rectopexy is 0.4%, for open rectopexy is 0.6%, and for perineal rectopexy is 1.2%. Additionally, bleeding risks are also lower, with reintervention rates for bleeding around 0.4%. However, the anatomical challenges and the need for precise fixation can still contribute to potential complications.

### Asymptomatic VTE in the postoperative setting

Composite VTE includes both symptomatic and asymptomatic VTE, providing a comprehensive measure in clinical trials (25). A recent multicenter cohort study involving solid cancer patients reported a composite VTE rate of 11.4% at 6 mo during anticoagulant therapy (26). Similarly, a prospective cohort study (the CRC-VTE study) in China observed a high composite VTE rate of 11.2% in CRC patients following surgery (27). The rate of symptomatic VTE in this study was 2.5%, consistent with the rates in randomized controlled trials (3). Two decades ago, the incidence of composite VTE events in China was reported to be higher (up to 38%) compared with now (28), primarily because of discontinuation of anticoagulant therapy due to bleeding concerns and surgeons' unawareness of VTE prophylactic guidelines and adherence to consensus treatment (29). Attention must be paid to timing of diagnosing asymptomatic VTE because regularly scheduled clinical and radiographic examinations are difficult to perform in post-discharge course in real-world settings.

The variation in VTE and bleeding risks across colorectal procedures underscores the need for a tailored approach. For surgeries with a high risk of VTE, a more aggressive prophylaxis regimen and closer post-discharge surveillance may be warranted. For low-risk procedures, standard prophylaxis may be sufficient. Surgeons must integrate this procedure-specific risk profile into their perioperative anticoagulation planning.

## Perioperative management strategies for DOAC in colorectal surgical patients

The perioperative management of patients who are receiving chronic DOAC therapy and require elective colorectal surgery is a common clinical scenario (30). DOACs exert their anticoagulant effects by inhibiting FXa (apixaban, rivaroxaban, and edoxaban) and thrombin directly (dabigatran), and are commonly used in the treatment of atrial fibrillation (AF) as well as in the prevention and treatment of VTE (31). The expected 30-d risk of postoperative VTE recurrence in patients with chronic anticoagulation can be categorized into three tiers: high risk >10%, moderate risk 4%–10%, and low risk <2% (32, 33). Similarly, those patients' risk of surgery-related bleeding can be empirically classified into high, low-to-intermediate, and minimal classes, with the expected 30-d postoperative major bleeding risk as ≥2%, 0%–2%, and ∼0%, respectively (32, 33). Upon this risk classification, colorectal surgery is considered to be a high bleeding risk procedure, necessitating adequate preoperative interruption of anticoagulants and delayed postoperative resumption to account for the longer time required for surgical site hemostasis (34).

To enhance clinical utility, we analyze FXa inhibitors (apixaban, rivaroxaban and edoxaban) and the direct thrombin inhibitor (dabigatran) separately in the following sections, given their distinct pharmacokinetic profiles and management considerations.

### Preoperative DOAC interruption

Current guidelines recommend a PK-based approach for preoperative interruption of DOAC in patients undergoing elective colorectal surgery. For high bleeding risk surgery, preoperatice DOAC interruption should be maintained for four to five half-lives (35). Elimination half-lives of FXa inhibitors are 8–12 h in patients with creatinine clearance (CrCl) above 30 mL/min (36). Dabigatran has a higher dependence on kidney clearance, so its elimination half-life is 10–14 h in patients with a CrCl at above 50 mL/min and 18–24 h in patients with a CrCl of 30–49.9 mL/min (36). Specifically, the preoperative interruption interval should correspond to 8–96 h depending on different types of DOACs and hepatic/renal functions of individuals to ensure minimal or no residual anticoagulant effect at the time of surgery (35, 36).

The aforementioned strategy was investigated in the PAUSE study, which is a cohort study of 3,007 patients with AF and a CrCl >30 mL/min who were undergoing elective surgical or nonsurgical procedures while on DOAC therapy (37). The 30-d postoperative incidence rates of symptomatic VTE events and major bleeding were as follows: 0.48% [95% confidence interval (CI): 0.16%–1.40%] and 1.35% (95% CI: 0.0%–2.0%) in the apixaban cohort; 0.30% (95% CI: 0.06%–1.68%) and 0.9% (95% CI: 0.0%–1.73%) in the dabigatran cohort; and 0.09% (95% CI: 0.02%–0.87%) and 1.85% (95% CI: 0.0%–2.65%) in the rivaroxaban cohort (37). A retrospective single-center study evaluated the perioperative management of 525 patients on DOAC therapy undergoing elective surgery or procedures. Unlike the PAUSE study, perioperative DOAC management in this study was not standardized and was left to the discretion of the attending physicians. Using this approach, 2.4% of patients experienced major bleeding, and 0.8% had thromboembolic events, which were higher incidence rates compared to those observed in the PAUSE study (38).

### Intraoperative bridging with low molecular weight heparin is not necessary

Compared to warfarin, the anticoagulant effect of DOACs decreases more rapidly after interruption and takes effect more quickly upon resumption of administration (39), thereby eliminating the need for low molecular weight heparin (LMWH) bridging during the perioperative DOAC interruption period. Studies have demonstrated that the perioperative management of patients undergoing noncardiac surgery while using DOACs without heparin bridging is safe and feasible (36). In a prospective registry of 901 patients undergoing elective surgery while on DOAC therapy, perioperative bridging with LMWH was associated with an increased risk of major bleeding (OR = 4.6; 95% CI: 1.6–13.2), while it had no significant impact on thromboembolic outcomes (OR = 1.9; 95% CI: 0.7–5.4) (40). In a meta-analysis, compared to the nonbridging group, perioperative bridging with LMWH was associated with a threefold higher incidence of major bleeding (4.8%; 95% CI: 3.4%–6.2% vs. 1.6%; 95% CI: 1.2%–2.0%), with no difference in the pooled incidence of stroke/systemic embolism (0.4%; 95% CI: 0.1%–0.9% vs. 0.3%; 95%CI: 0.1%–0.4%) (41). Notably, Fujikawa et al. observed that, in patients undergoing elective gastrointestinal surgery, the incidence of postoperative major bleeding was significantly higher in those treated with DOACs along with heparin bridging compared to those treated with warfarin or DOACs alone (14.7% vs. 4.8% vs. 1.4%, *P* = 0.011) (42). Nevertheless, in selected high thrombotic risk patients (e.g., recent VTE within 3 months, mechanical heart valves, or prior thromboembolism during anticoagulation interruption), individualized consideration of bridging therapy may be warranted after multidisciplinary evaluation.

### Postoperative DOAC resumption

The timing of postoperative resumption of DOACs is based on the assessment of bleeding risk associated with surgery and hemostasis at the surgical site, including blood loss through surgical dressings and drains. Colorectal surgery is considered high-risk for bleeding, and guidelines recommend restarting DOACs 2 d after surgery (43). Flexibility in the timing of postoperative DOAC resumption is necessary, as the anticoagulant effect peaks 2–3 h after administration, which may increase the risk of postoperative bleeding, particularly relevant if patients experience greater than expected postoperative blood loss (36). If postoperative bleeding occurs or hemostasis at the surgical site is uncertain, resumption should be delayed (36).

In the PAUSE study, DOAC resumption was not initiated earlier than 48–72 h after surgery, with a high bleeding risk, which was associated with a 30-d postoperative major bleeding rate of 2.49% (95% CI: 0%–4.25%) in this population (37). Even in a subgroup of patients undergoing radical prostatectomy (identified as procedure with high thromboembolism risk and bleeding risk), the strategy of PAUSE achieved total rates of thromboembolic/bleeding complications within 30 d postoperatively as low as 3.7% (44).

Despite these advantages, anticoagulants remain the leading cause of emergency department visits due to adverse drug events, accounting for ∼14.90% (95% CI: 10.70%–19.10%) of all ADE-related visits in the USA from 2017 to 2019 (45). Clinical research over the past decade has revealed significant interpatient variability in DOAC plasma levels, raising concerns about the appropriateness of a “one size fits all” dosing strategy for DOACs (46, 47).

### Laboratory assays and perioperative monitoring of DOAC levels

Quantitative measures for the direct assessment of DOACs' effects involve anti-FXa activity (for FXa inhibitors), dilute thrombin time (for dabigatran), ecarin thrombin time (for dabigatran), and drug plasma levels (48, 49). For the anti-FXa assay, FXa is added to plasma containing a FXa substrate (e.g., heparin) that is tagged with a chromophore. When the chromophore is cleaved by FXa, a color change results that is directly proportional to the levels of FXa present in the assay (50). The chromogenic anti-FXa assay for the anticoagulant effects of FXa inhibitors is precise, sensitive, and accurate with a plasma levels-dependent inhibition of FXa activity (correlation coefficient of 0.9669) (50). No effect is expected on the anti-FXa assay from dabigatran based on the mechanism of direct thrombin inhibition. Dabigatran prolongs the dTT in a linear dose-relationship and can accurately predict anticoagulation intensity (51). The dTT demonstrated a high correlation with dabigatran plasma levels when used with both in-house and Hemoclot-derived dabigatran calibrators (correlation coefficients of 0.9981 and 0.9982, respectively) (52). The ECT directly measures thrombin generation, with a correlation coefficient of 0.92 with dabigatran plasma levels (53). No effect is expected on dTT and ECT from this drug class based on the mechanism of direct FXa inhibition.

Few data exist regarding the effect of measuring preoperative DOAC levels on perioperative bleeding risk. Some have advocated that a preoperative DOAC level less than 50 ng/mL is a safe threshold to allow surgery to proceed, as it considered a minimal, clinically insignificant anticoagulant effect. And a DOAC level less than 30 ng/mL may be considered an undetectable level (54). For colorectal surgery, patients with multiple factors (e.g., age ≥75 years or renal insufficiency) interfering with PK of DOACs, or patients with uncertain time windows, may benefit from low residual DOAC levels, particularly for procedures associated with a high risk of bleeding (e.g., total proctocolectomy), where minimal or no anticoagulant effect is desired during surgery (37). Some studies have assessed preprocedural DOAC levels and clinical factors associated with higher residual levels (55, 56). A secondary analysis of PAUSE study was performed to identify risk factors associated with unsatisfied residual DOAC levels (55). In low-risk procedures, age ≥75 years, female sex, and CrCl >50 mL/min were associated with both DOAC levels ≥30 ng/mL and ≥50 ng/mL. Additionally, a DOAC interruption of 36 h was linked to levels ≥30 ng/mL, while standard DOAC dosing was associated with levels ≥50 ng/mL. For high-risk procedures, weight <70 kg, CrCl >50 mL/min, and standard DOAC dosing were associated with residual DOAC levels ≥30 ng/mL, whereas female sex was linked to levels ≥50 ng/mL (55). The CORIDA study, a prospective observational study involving 422 DOAC-treated patients undergoing elective procedures, measured preprocedural DOAC levels. Unlike the PAUSE study, it did not standardize perioperative DOAC management, resulting in a wide variation in DOAC interruption duration (1–218 h). The study identified the duration of DOAC interruption, CrCl <50 mL/min, and antiarrhythmic drug use as predictors of preprocedural DOAC levels ≥30 ng/mL, while patient age, sex, and weight were not significant predictors (56).

Meanwhile, the feasibility and cost-effectiveness of DOAC level monitoring remain important practical considerations. While the anti-FXa assay is becoming more accessible, its widespread implementation is constrained by factors including specialized laboratory infrastructure, trained personnel, and the limited availability of DOAC-specific calibrators. A survey of 46 specialized coagulation assays revealed that only 39% offered anti-Xa assays for rivaroxaban and 22% offered this assay for apixaban (57). Current guidelines applicable to China generally recommend a PK-based management strategy without mandatory preprocedural DOAC level measurement (58). This approach prioritizes a standardized interruption interval based on the drug's half-life, renal function, and procedural bleeding risk, reserving laboratory assessment for complex or high-risk scenarios (e.g., emergency surgery, severe renal impairment, or suspected overdose). Within this framework, greater emphasis is placed on the safety thresholds and interruption timelines. For elective surgery, a preprocedural DOAC level below 50 ng/mL is widely considered to represent a minimal anticoagulant effect associated with bleeding risk, while a level below 30 ng/mL is often regarded as negligible (54). To achieve this, a preoperative interruption period corresponding to four to five drug half-lives is recommended for procedures with high bleeding risk.

### DOAC management in emergency colorectal surgery

Patients treated with DOACs who require emergency surgery have a high risk of bleeding (17%–23%) and VTE (7%–16%) (59). Management decisions for patients taking DOACs who need emergency colorectal surgery involve multiple patient- and procedure-related factors, making it difficult to develop standardized management protocols (60, 61). The anticoagulant effect of a DOAC can be neutralized with DOAC specific reversal agents, including andexanet-α for apixaban, edoxaban and rivaroxaban or idarucizumab, a monoclonal antibody fragment that act as a specific reversal agent for dabigatran (62). Prothrombin complex concentrate (PCC) and activated PCC, which are nonspecific prohemostatic agents, can be used to reverse the effect of all DOACs (62). If DOAC level testing is available, a DOAC level at or above 50 ng/mL may necessitate the use of a DOAC reversal agent, whereas a level less than 50 ng/mL may allow the operation to proceed without a reversal intervention (63). If DOAC level testing is unavailable, DOAC reversal agent should be considered if the most recent DOAC dose was taken less than 48 h before the procedure (60).

### Perioperative DOAC management of high-risk population

Inflammatory bowel disease (IBD) is a chronic inflammatory disorder associated with a markedly prothrombotic state (64). The underlying inflammation elevates the risk of thromboembolism by 2–3 fold (65). This risk further amplified by active IBD disease, flares-up, surgery, steroid treatment, and hospitalization (66). Meanwhile, IBD patients may suffer gastrointestinal bleeding, especially those with ulcerative colitis (67). Anticoagulant therapy may increase the bleeding risk in IBD patients, which may consequently influence treatment decisions (68). For IBD patients on DOACs, a standardized PK-based interruption strategy is recommended (69). However, in patients with active severe colitis, recent significant gastrointestinal bleeding, or undergoing surgery involving extensive mucosal resection, a more conservative approach may be considered in consultation with a gastroenterologist and hematologist (69). Postoperative DOAC resumption should generally follow the high bleeding risk protocol (e.g., 48–72 h post-surgery after hemostasis is secured) (69). Bridging therapy is not routinely recommended due to its associated bleeding risk (70).

Frailty constitutes a high-risk category for perioperative DOAC management due to its amplification of both thrombotic and hemorrhagic complications (71). Frail patients present a complex challenge characterized by multimorbidity, polypharmacy, and reduced physiological reserve (72). Of particular concern is the heightened susceptibility to bleeding due to factors such as increased fall risk, acute kidney injury, and prevalent drugs interactions (73). Prolonged immobility and delayed recovery may extend the window of vulnerability to VTE (73). Therefore, a standard or more conservative DOAC interruption strategy is warranted. Preoperative interruption should follow PK timelines for high bleeding risk surgery (74). Postoperative resumption should be carefully timed, balancing thrombotic therapy needs against the heightened risk of spontaneous or traumatic hemorrhage (75). Factors such as dynamic renal function, nutritional status, and cognitive function must be serially evaluated to guide safe restarting of therapy.

Renal function is a critical determinant of DOACs clearance, particularly for dabigatran (∼85% renal excretion), and to a significant extent for edoxaban, rivaroxaban, and apixaban (76). Impaired renal excretion prolongs the half-life and elevates plasma levels, thereby increasing the risk of major bleeding during the perioperative period (76). For patients with moderate to severe renal impairment (e.g., CrCl 15–50 mL/min), a prolonged preoperative DOAC interruption period is mandatory. The duration must be tailored to the specific agent and the degree of renal dysfunction, often extending 24–48 h beyond the standard window recommended for normal renal function (77). In urgent situations or for patients with fluctuating renal function, laboratory monitoring can objectively assess residual anticoagulant activity prior to surgery (46, 63). Postoperative resumption must be highly individualized. Dose adjustment according to the guidelines based on current renal function is imperative upon restarting (76).

## Variability in DOAC plasma levels and their exposure–effect relationship

DOACs exhibit relatively predictable dose–exposure (PK) and dose–response (PD) relationships. However, when fixed doses of DOACs are administered, some patients may exhibit drug levels deemed too high or too low, demonstrating significant interindividual variability in plasma levels (78). According to guidelines, among patients receiving an appropriate normal dose of DOACs, 9% have levels below the expected range, and 23.8% have levels above the expected range (79). A study involving 152 patients using DOACs assessed intraindividual and interindividual variability by calculating the coefficient of variation (CV). The study found that, for interindividual variability, patients on the recommended dose had trough CVs ranging from 48% to 81% and peak CVs ranging from 25% to 69%; intraindividual variability was lower, with trough CVs ranging from 18% to 33% and peak CVs from 15% to 29% (80). A real-world study specifically targeting older patients found up to 48-fold and 13-fold variations in trough and peak levels, respectively (78). Compared to patients taking rivaroxaban or dabigatran, a significantly higher proportion of patients taking apixaban had peak levels within the reported range (82.9% vs. 44.3% vs. 64.3%, respectively; *P* < 0.001). One-third of the variability in DOAC levels is attributed to the effects of DOAC dose, renal function, and gender (78). A retrospective cohort study of trauma patients found considerable variability in anti-FXa levels among those using rivaroxaban or apixaban; older, smaller females with decreased renal function exhibited higher DOAC-specific anti-FXa levels post-trauma (81).

In order to balance risk of anticoagulation underuse and anticoagulation-related bleeding, several investigator-initiated studies have explored the exposure–effect relationship of DOACs for individualized patient dosimetric estimation. Results of those studies and target range of different DOACs are given in Table 1 (80–90), and the main findings are summarized below. Plasma levels outside the expected range are associated with higher incidences of adverse events, and some form of therapeutic drug monitoring may improve patient outcomes (82–92).

**Table 1:** The peak and trough levels of DOACs at different doses, and their exposure-effect relationship.

DOACs	Population	Dose	Peak levels (ng/mL)	Trough levels (ng/mL)	Exposure-effect relationship	Ref.
Rivaroxaban	AF	15 mg qd	344.25 (25th–75th, 222.75-562.5)	29.25 (25th–75th, 11.25-74.25)	Peak levels (HR = 2.07 per 1.0 IU/mL; 95% CI: 1.18-3.65) for hemorrhagic events	Wada et al. (82)
		10 mg qd	279 (25th–75th, 153–384.75)	11.25 (25th–75th, 2.25–63)		
	AF	20 mg qd	266 (min-max, 142–552)	38 (min-max, 5–125)	Trough levels were higher in patients with bleeding than in patients without it (48 ± 30 vs 34 ± 26, *P* = 0.02)	Miklič et al. (83)
		15 mg qd	214 (min-max, 152–530)	37 (min-max, 10–141)		
	VTE	20 mg qd	270 (5th–95th, 189–419)	26 (5th–95th, 6–87)	NA	Mueck et al. (84)
	VTE	15 mg qd	339.75 ± 243	103.5 ± 162	NA	Ono et al. (85)
		30 mg qd	661.5 ± 258.75	351 ± 276.75		
Apixaban	AF	5 mg bid	659.25 (25th–75th, 490.5–832.5)	405 (25th–75th, 254.25–524.25)	Tough levels (HR = 2.22 per 1.0 IU/mL; 95% CI: 1.16–4.24); Peak levels (HR = 2.47 per 1.0 IU/mL; 95% CI: 1.28–4.75) for hemorrhagic events	Wada et al. (82)
		2.5 mg bid	432 (25th–75th, 326.25–618.75)	261 (25th–75th, 175.5–507.45)		
	AF	2.5 mg bid 5 mg bid	217 (min-max, 45–658)	111.3 (min-max, 22–515)	NA	Testa et al. (86)
	VTE	2.5 mg bid	90 (5th–95th, 37–161)	34 (5th–95th, 7–68)	NA	Reda et al. (87)
		5 mg bid	160 (5th–95th, 63–299)	71 (5th–95th, 13–114)		
	VTE	5 mg bid	542.3 ± 258.8	391.5 ± 240.75	NA	Ono et al. (85)
		10 mg bid	920.3 ± 265.5	663.8 ± 324		
Edoxaban	AF	60 mg qd	NA	36.1 (25th–75th, 19.4–62.0)	NA	Ruff et al. (88)
		30 mg qd	NA	27.0 (25th–75th, 14.6–44.6)		
		15 mg qd	NA	12.4 (25th–75th, 7.3–21.0)		
	VTE	30 mg qd	281.25 (25th–75th, 173.25–382.5)	24.75 (25th–75th, 15.75–51.75)	Peak levels ≥ 2.09 IU/mL (HR = 11.5, 95% CI: 1.3–105.2) for MB and CRNMB	Nakano et al. (89)
	VTE	30 mg qd	166.5 ± 87.75	42.8 ± 31.5	NA	Ono et al. (85)
		60 mg qd	317.25 ± 155.25	38.25 ± 29.25		
Dabigatran	AF	110 mg bid	96.03 (10th–90th, 38.43–191.90)	38.46 (10th–90th, 13.02–108.11)	No significant relationship between peak level and bleeding or stroke/SEE (*P* = 0.169, 0.430)	Zhu et al. (90)
	AF	110 mg bid	150.5 (10th–90th, 51.3–425.6)	77.6 (10th–90th, 28.4–292.8)	Trough levels ≥ 243.9 ng/mL (HR = 8.0, 95% CI: 3.0–25.0) for hemorrhagic events	Chaussade et al. (91)
	AF	110 mg bid 150 mg bid	157 (min-max, 36–633)	78 (min-max, 36–324)	Peak levels ≥ 186 ng/mL (OR = 2.7, 95% CI: 1.3–5.4) for hemorrhagic events	Testa et al. (92)

DOACs, direct oral anticoagulants; AF, atrial fibrillation; HR, hazard ratio; 95%CI: 95% confidence interval; VTE, venous thromboembolism; qd once daily; bid, twice daily; CRNMB, clinically relevant nonmajor bleeding; MB, major bleeding; SEE, systemic embolic event; OR, odds ratio.

^a^ Hemorrhage included major bleeding, clinical relevant nonmajor bleeding, and minor bleeding NA, not available.

The documented interindividual variability in DOAC plasma levels and its established exposure-effect relationship carry significant implications for perioperative management in colorectal surgery. In clinical practice, this translates to tailoring the timing of preoperative DOAC interruption, particularly for patients at the extremes of these covariates (e.g., the elderly, those with renal impairment, or low body weight), to ensure adequate drug clearance. Conversely, awareness of factors associated with low drug levels can inform decisions to minimize the duration of subtherapeutic anticoagulation in high thrombotic risk individuals.

## Pharmacogenomics of DOACs

In light of the rapidly growing understanding of the complex human genome and its derived functional biology allows selection of candidate single nucleotide polymorphisms (SNPs) based on the current knowledge of pharmacogenomics to optimize the safety and efficacy of anticoagulant therapy.

The pharmacogenomics of DOACs remains an emerging and increasingly popular area of interest, with the majority of research focusing on genes that influence the metabolic pathways of DOACs. Most DOACs follow a comparable metabolic pathway (93), involving absorption in the gastrointestinal tract by enterocytes through active and passive transport mechanisms, followed by entry into the hepatic portal circulation. In the liver, DOACs that are prodrugs, such as dabigatran etexilate, are converted to their active forms by specific enzymes, including carboxylesterase (CES)1 (94). These active compounds then enter the systemic circulation to exert their anticoagulant effects or are eliminated from the body by ABC efflux transporters, including P-glycoprotein (P-gp) (encoded by the *ABCB1* gene) and proteins encoded by *ABCG2*. Some DOACs, including edoxaban, require the organic anion transporter protein (OATP)1B1 (encoded by *SLCO1B1*) for partial hepatic uptake. Following this, the active drug enters the systemic circulation to exert its pharmacological effects (95). Elimination of DOACs occurs via hepatic and renal pathways. Specifically, dabigatran is activated hepatically and then cleared predominantly by the kidneys (85%). In contrast, FXa inhibitors undergo hepatic metabolism primarily via CYP3A4 and CYP3A5, with rivaroxaban additionally metabolized by CYP2J2 (94, 95). Edoxaban is primarily metabolized by CES1 and, to a lesser extent, by CYP3A4/3A5, and is transported by P-gp (95). The proportion of the FXa inhibitors not cleared by the liver, is excreted by the kidneys (33%, 27%, and 35%, respectively) (95–97).

Polymorphisms in genes encoding these metabolic enzymes and transporters can lead to alterations in the pharmacokinetics of DOACs and influence clinical outcomes such as bleeding risk. The key genetic polymorphisms associated with each DOAC, their impact on plasma levels, and their reported associations with clinical bleeding outcomes are systematically summarized in Table 2 (98–105).

**Table 2:** Pharmacogenomic variants affecting the pharmacokinetics of DOACs.

DOACs	SNPs	Grouped Genotypes	Plasma Levels (ng/mL)	*P*-value	Clinical Outcomes	*P*-value	Ref.
Rivaroxaban	*ABCB1*						
			Peak levels		Hemorrhage^a^		
	rs2032582	AA (47.22%) GA/GG (52.78%)	213.34 ± 100.95 239.86 ± 150.92	<0.001**	OR=2.262 (95% CI: 1.188–4.310)	0.013*	Zhang et al. (98)
			Trough levels		CRNMB		
	rs1045642	CC (17.2%) CT/TT (82.8%)	57.7 (25th-75th, 23.3–75.8) 56.5 (25th-75th, 36.5–76.5)	0.501	OR=5.574 (95% CI: 4.408–7.040)	<0.001**	Sychev et al. (99)
			Trough levels		CRNMB		
	rs4148738	CC (28.9%) CT/TT (71.1%)	57.7 (25th-75th, 28.3–98.0) 53.4 (25th-75th, 33.9–72.9)	0.481	OR=3.200 (95% CI: 2.790–3.670)	<0.001**	Sychev et al. (99)
			Trough levels		Hemorrhage^a^		
	rs1128503	CC (17.42%) CT/TT (82.58%)	20.23 (25th-75th, 13.64–51.70) 30.47 (25th-75th, 16.47–61.00)	0.04*	OR=1.30 (95% CI: 0.85–1.99)	0.23	Wang et al. (100)
Apixaban	*ABCG2*						
			Trough levels				
	rs2231142	CC (45.4%) CA/AA (54.6%)	129.52 (25th-75th, 88.35–170.70) 148.85 (25th-75th, 81.72–207.16)	0.003**	NA	NA	Ueshima et al. (101)
	*CYP3A5*						
			Trough levels				
	rs776746	AA (6.8%) AG/GG (93.2%)	87.59 (25th-75th, 73.37–114.55) 141.36 (25th-75th, 96.12–190.62)	0.006**	NA	NA	Ueshima et al. (101)
Edoxaban	*SCLO1B1*						
					Hemorrhage^a^		
	rs4149056	TT (14%) CT/CC (86%)	NA	NA	OR=4.26 (95% CI: 1.55–11.69)	0.003**	Han et al. (102)
			Peak levels				
	rs4149056	TT (83.8%) CT/CC (16.2%)	255.1 ± 97.4 268.0 ± 94.7	0.62	NA	NA	Vandell et al. (103)
			Trough levels				
	rs4149056	TT (83.8%) CT/CC (16.2%)	12.3 ± 5.4 11.6 ± 4.8	0.61	NA	NA	Vandell et al. (103)
Dabigatran	*ABCB1*						
			Peak levels		Major bleedings		
	rs1045642	CC (25%) CT/TT (75%)	124.1 (25th-75th, 79.9–177.7) 214.5 (25th-75th, 135.8–294.4)	<0.001**	OR=2.90 (95% CI: 1.17–7.19)	0.022*	Sychev et al. (104)
	*CES1*						
			Trough levels		Minor bleedings		
	rs2244613	CC (38.4%) CA/AA (61.6%)	76.1 ± 43.1 82.0 ± 35.0	<0.001**	OR=2.71 (95% CI: 1.05–7.00)	0.034*	Ji et al. (105)

DOACs, direct oral anticoagulants; *ABCB1*, ATP Binding Cassette Subfamily B Member 1; OR, odds ratio; 95%CI, 95% confidence interval; CRNMB, clinically relevant nonmajor bleeding; *ABCG2*, ATP Binding Cassette Subfamily G Member 2; *CYP3A5*, Cytochrome P450 Family 3 Subfamily A Member 5; *SCLO1B1*, Solute Carrier Organic Anion Transporter Family Member 1B1; *CES1*, Carboxylesterase 1; NA, not available.

^a^ Hemorrhage included major bleeding, clinical relevant nonmajor bleeding, and minor bleeding.

* *P* < 0.05.

** *P* < 0.01.

While routine genotyping is not currently standard practice, pharmacogenomic information holds promise for personalizing DOAC therapy in high risk or complex colorectal surgical patients. For example, identifying a patient scheduled for total proctocolectomy who carries the ABCB1 rs1045642 CT/TT genotype might prompt a more cautious approach with rivaroxaban, with a longer preoperative interruption or preoperative anti-FXa monitoring. As evidence grows and testing becomes more accessible, integrating pharmacogenomics with clinical factors could refine risk prediction and optimize dosing, moving towards precision antithrombotic management.

## Genetic risk score for patients on DOACs

The development of genetic risk scores (GRSs) has emerged as a crucial step toward personalized anticoagulant therapy. By integrating multiple SNPs with or without clinical factors, GRSs aim to quantify an individual's genetic predisposition towards thrombotic or bleeding events, thereby refining risk stratification beyond clinical parameters alone. A series of studies has demonstrated that, by integrating SNPs with clinical factors, constructed models can be used to predict the risk of a first VTE in various populations (106–109). However, predictive models for estimating thromboembolic events recurrence risk or evaluating bleeding risk are preferred, in order to guide DOAC therapy duration and optimize dosing regimens (110). Key examples of developed predictive models, including their constituent variables (clinical and genetic), modeling methods, and performance metrics are systematically summarized in Table 3 (102, 109, 111–117). These models span different clinical targets: predicting VTE recurrence (e.g., DAMOVES score, L-TRRiP score) (111, 113), predicting first VTE in cancer patients (e.g., TiC-Onco and ONCOTHROMB scores) (109, 113), and most relevantly, predicting bleeding risk in patients treated with DOACs (102, 114–117). The studies on bleeding risk prediction in DOAC patients highlight the incremental value of adding genetic variants (e.g., ABCG2, ABCB1, SLCO1B1, RYR2) to clinical models, often improving the model's discriminative ability (102, 114–117).

**Table 3:** The development of genetic risk scores.

Ref.	Model	Variables Included		Model fitting method	AUROC (95% CI)	Hosmer–Lemeshow test
		Clinical variables	Genetic variables			
Predict the risk of VTE recurrence						
Franco Moreno et al. (111)	DAMOVES score	Age, sex, BMI, varicose veins, D-dimer, FVIII levels	*F5* rs6025*, F2* rs1799963	Cox regression	0.91 (95%CI is not shown)	NA
Timp et al. (112)	L-TRRiP score	Sex, type and location of first VTE, surgery, pregnancy/puerperium, hormone use, plaster cast, immobility in bed, history of cardiovascular disease	*F5* rs6025*, F2* rs1799963, *ABO* rs8176719, *FGG* rs2066865, *F11* rs2036914	Cox regression	0.70 (0.68–0.73)	NA
Predict the risk of first VTE						
Muñoz Martín et al. (113)	TiC-Onco score	BMI, family history, primary tumor site, tumor stage	*SERPINA10* rs2232698, *F5* rs6025, *F13A1* rs5985, *F5* rs4524	Logistic regression	0.73 (0.67–0.79)	NA
Muñoz et al. (109)	ONCOTHROMB score	BMI, tumor site, tumor stage	*F5* rs4524, *F5* rs6025, *SERPINA10* rs2232698, *SERPINE1* rs2227631, *LPL* rs268, *HIVEP1* rs169713, *RSPO4* rs11696364, *APOA4* rs5110, *F13B* rs6003	Logistic regression	0.781 (0.735–0.822)	NA
Predict the risk of bleeding in patients treated with DOACs						
Yoon et al. (114)	Model Ⅱ	Sex, age, overdose, rivaroxaban, anemia	*ABCB1* rs3842, *APOB* rs693, *APOB* rs13306198	Logistic regression	0.72 (0.65–0.80)	*χ*^2^ = 0.63 *P* = 0.73
Kim et al. (115)	Model Ⅰ	History of bleeding, concurrent use of PPI	*ABCG2* rs3114018, *ABCB1* rs1045642	Logistic regression	-	χ^2^ = 5.121 *P* = 0.163
	Model Ⅱ	Modified HAS-BLED score, concurrent use of PPI	*ABCG2* rs3114018, *ABCB1* rs1045642	Logistic regression	-	χ^2^ = 4.947 *P* = 0.763
Han et al. (102)	Model Ⅰ	Sex, age, prescription dose	*ABCB1* rs3842, *SLCO1B1* rs999278, *SLCO1B1* rs2306283, *SLCO1B1* rs4149056, *SLCO1B1* rs2417957	Logistic regression	0.79 (0.67–0.91)	χ^2^ = 0.11 *P* = 0.991
	Model Ⅱ	Sex, age, prescription dose	*ABCB1* rs3842, *SLCO1B1* rs4149057, *SLCO1B1* rs2306283, *SLCO1B1* rs4149056, *SLCO1B1* rs2417957	Logistic regression	0.78 (0.65–0.91)	χ^2^ = 2.34 *P* = 0.674
	Model Ⅲ	Sex, age, prescription dose	*ABCB1* rs3842, *SLCO1B1* rs999278, *SLCO1B1* rs2417957, SLCO1B1*15	Logistic regression	0.78 (0.64–0.91)	χ^2^ = 2.43 *P* = 0.657
	Model Ⅳ	Sex, age, prescription dose	*ABCB1* rs3842, *SLCO1B1* rs4149057, *SLCO1B1* rs2417957, SLCO1B1*15	Logistic regression	0.78 (0.65–0.91)	χ^2^ = 2.32 *P* = 0.677
Jang et al. (116)	Model Ⅱ	Age, sex, overdose, rivaroxaban, anemia, creatinine clearance	*ABCB1* rs3842, *RYR2* rs10925391, *RYR2* rs12594, *RYR2* rs17682073, *RYR2* rs3766871, *RYR2* rs6678625	Logistic regression	0.803 (0.735–0.871)	χ2 = 4.950 *P* = 0.550
Yee et al. (117)	Model Ⅰ	Age, creatinine clearance	*AGT* rs5050, *ACE* rs4353	Logistic regression	0.738 (0.649–0.826)	χ^2^ = 2.388 *P* = 0.935

BMI, body mass index; AUROC, the area under the receiver operating characteristic curve; 95% CI, 95% confidence interval; VTE, venous thromboembolism; *ABCB1*, ATP binding cassette subfamily B member 1; *APOB*, apolipoprotein B; TIA, transient ischemic attack; PPI, proton pump inhibitor; *ABCG2*, ATP Binding Cassette Subfamily G Member 2; *SLCO1B1*, solute carrier organic anion transporter family member 1B1; *RYR2*, ryanodine receptor 2; *AGT*, angiotensinogen; *ACE*, angiotensin I-converting enzyme.

For the perioperative management of colorectal surgical patients on DOACs, especially those with IBD, frailty, or renal impairment, the incorporation of the GRS may serve as a valuable instrument for enhancing individualized decision-making. These high-risk populations often present with complex and competing risks of thrombosis and bleeding, which may not be fully captured by clinical scores alone. A validated GRS could complement existing risk assessments by quantifying a patients' inherent genetic predisposition toward bleeding or thrombotic events. For example, in IBD patients with active mucosal inflammation who plan to undergo colectomy, a GRS indicating elevated bleeding risk might support extending the preoperative DOAC interruption window or guiding postoperative resumption timing. Similarly, in frail patients or those with renal impairment, GRS data could help personalize the duration of anticoagulation interruption and inform dose adjustment upon resumption, thereby balancing proceducal safety with ongoing thromboembolic protection.

## Regional variations in anticoagulation management in surgical patients

The management of anticoagulation in surgical patients exhibits notable differences between continental China and the Western world, shaped by distinct epidemiological patterns, genetic backgrounds, and clinical practices. The incidence of VTE among cancer surgical patients is significantly lower in China (1.85–9.88 per 1,000 person-years) compared to Western cohorts (e.g., up to 58 per 1,000 person-years in the UK) (118, 119). Even in high-risk surgical settings such as colorectal cancer surgery, the reported VTE rate within one month postoperatively is 11.2% in China, with a majority being asymptomatic, whereas Western data often reflect higher symptomatic rates (27). This lower observed burden may be attributed to the absence of common thrombophilic mutations (e.g., Factor V Leiden, prothrombin G20210A) in Asian populations, alongside lifestyle factors such as lower obesity rates and traditional diets (120). However, potential under diagnosis in Asia due to lower clinical suspicion and limited routine screening may also contribute to the reported disparity (121).

Bleeding risks under anticoagulation also revealed regional differences. Chinese patients demonstrate higher susceptibility to bleeding with vitamin K antagonists (VKAs), largely due to pharmacogenetic variants such as VKORC1 (122). However, real-world data suggest that DOACs, particularly apixaban, are associated with a lower risk of gastrointestinal bleeding in Asian patients (123). Clinical practices further underscore these regional disparities. Thromboprophylaxis is underutilized in China, with surveys indicating that a significant proportion of surgeons do not routinely administer it (124).

These disparities highlight the critical need for tailored, region-specific anticoagulation strategies that carefully balance thromboprophylaxis efficacy against bleeding risks, integrating genetic, environmental, and healthcare system factors into clinical decision-making.

## Future perspectives

The future of perioperative management of colorectal surgical patients receiving DOACs lies in addressing the increased risk of major bleeding, particularly in patients undergoing high bleeding risk procedures. Assessing residual DOAC levels in patients with high risk factor (e.g., renal insufficiency) at the time of invasive procedures and their correlation with bleeding events will provide valuable insights. Exploring genetic factors that influence the risk of recurrent VTE or bleeding events during DOACs treatment will allow for more tailored and effective anticoagulant management. However, current genetic research is predominantly based on European populations, limiting its applicability to other ethnic backgrounds (111). Specifically, future directions should include: (1) Prospective randomized trials directly comparing different DOACs management strategies (e.g., standardized vs. personalized interruption) in colorectal surgery; (2) Development and validation of integrated risk prediction models that combine clinical, surgical, laboratory, and genetic data for both VTE and bleeding; (3) Cost-effectiveness analyses of routine perioperative DOAC level monitoring and pharmacogenomic testing in high risk subgroups; (4) Multicenter studies in Asian and other underrepresented populations to define enthnicity-specific pharmacogenomic profiles and management algorithms.

## Conclusion

This review highlights the complexity of managing colorectal surgical patients receiving DOACs. The variable risks of VTE and bleeding necessitate an individualized approach that considers patient-specific factors, such as renal function, drug plasma levels, and timing of anticoagulant interruption and resumption. A PK-based strategy, which tailors the preoperative and postoperative management to minimize residual DOAC effects, appears critical for improving outcomes. Moreover, emerging pharmacogenomic insights show promise in refining risk prediction and guiding personalized dosing regimens. Future research should aim to elucidate the genetic factors affecting DOAC metabolism and response, particularly in diverse populations, and to validate innovative laboratory methods for monitoring anticoagulant activity. Integrating clinical, pharmacologic, and genetic data will be essential in developing more effective, evidence-based strategies to reduce adverse events and optimize the perioperative care of these patients.
